# Effect of Fe_3_O_4_ nanoparticles on germination of seeds and concentration of elements in *Helianthus annuus* L. under constant magnetic field

**DOI:** 10.1038/s41598-020-64849-w

**Published:** 2020-05-15

**Authors:** Krzysztof Kornarzyński, Agnieszka Sujak, Grzegorz Czernel, Dariusz Wiącek

**Affiliations:** 1 Department of Biophysics, University of Life Sciences in Lublin, Akademicka 13, 20-933 Lublin, Poland; 2 Institute of Agrophysics, Polish Academy of Sciences, Doświadczalna 4, 20-290 Lublin, Poland

**Keywords:** Biophysics, Plant sciences

## Abstract

The aim of the study was to investigate the effect of the Fe_3_O_4_ nanoparticles (Fe-NPs) on the germination of sunflower seeds, early growth of seedlings and the concentration of selected elements in seedlings. The influence of constant magnetic fields in systems with and without Fe-NPs was investigated. Experiments were done on seeds subjected to germination under constant magnetic field (0 (control), 5, 25 and 120 mT) for 7 days in the presence of solution containing 0, 50 or 500 ppm Fe-NPs. No significant effect of Fe-NPs and the magnetic field on germination of seeds and the growth of seedlings has been demonstrated. In most cases, a decrease in germination parameters was observed. For the majority of samples the relative decrease in the concentrations of elements was demonstrated mainly for samples without Fe-NPs. Interestingly, a significant decrease in the concentrations of trivalent (including iron - Fe) and toxic elements in samples containing Fe-NPs in relation to control samples was observed. The authors suggest that in this case the binding (adsorption) of these elements in the roots and seeds of the sunflower by Fe-NPs took place. This explains the lower iron content in seedlings than in seeds prior to sowing.

## Introduction

Nanoparticles (NPs) have become a very attractive research object due to their unique physicochemical properties. There are many possibilities of their applications in industry, technology^1^ and medicine^2,3^ as well as in agriculture^4^. Agricultural applications include fertilizers and pesticides used to control pests or diseases^5^ as well as sensors for monitoring soil and plant quality^6^. Nano-layers of macro- and micronutrients or nano-carriers with nutrients are also used to enhance plant growth and protection^7^.

The effect of NPs on plants depends on their composition, concentration, size and physical properties as well as on plant species^8^. Rizwan *et al*.^9^ studied the effect of phosphorus nanoparticles (P-NPs) on cultivated plants whereas Siddiqui and Al-Whaibi^10^ applied SiO_2_ nanocrystals to tomato, both obtaining positive effects on plant growth, yield, mineral nutrition and photosynthesis. Ag-NPs influenced the mitotic index and root growth of *Vicia faba* seedlings^11^ and sorghum (*bicolor Sorghum*) and had growth-inhibiting effects depending on the concentration of NPs^12^. Mahmoodzadeh *et al*.^13^ treated canola seeds, Feizi *et al*.^14^ fennel seeds and Samadi *et al*.^15^ peppermint seeds (*Mentha piperita*) with TiO_2_-NPs and observed an increase in root length and a positive effect on the concentration of photosynthetic pigments.

The effect of toxicity was studied and observed for aluminum oxide (Al_2_O_3_), silicon dioxide (SiO_2_), magnetite (Fe_3_O_4_) and zinc oxide (ZnO) nanoparticles on *Arabidopsis thaliana*^16^ showing the highest phytotoxicity for ZnO-NPs. The influence of NPs (multilayer carbon nanotubes, aluminum, alumina, zinc and zinc oxide) on seed germination and plant roots growth (radish, rapeseed, ryegrass, lettuce, corn and cucumber) was investigated^17^. No NPs effect on germination of seeds was noted, however nano-Zn and nano-ZnO suspensions caused elongation of the roots of the studied plant species.

Zhu *et al*.^18^ investigated the effect of Fe_3_O_4_-NPs on pumpkins (*Cucurbita maxima*) and found that they can absorb, displace and accumulate nanoparticles in tissues.

The application of super paramagnetic iron oxide NPs (SPION)^17^ resulted in the increased chlorophyll levels in soybean leaves. Spherical Fe0-NPs and Fe_3_O_4_-NPs caused the inhibition of germination and leaf extension in soft wheat (*Triticum vulgare* Vill.)^19^. Fe-NPs positively influenced the germination and growth of wheat seedlings during exposure to drought and excessive salt - an increase in the weight and length of seedlings, shoots and roots was observed^20^.

Shrivastava *et al*.^21^ and Das *et al*.^22^ used aqueous suspension of non-paramagnetic nanoparticles of iron pyrite of (FeS_2_ + H_2_O) for the treatment of spinach seeds. In both cases aqueous suspension of nano iron pyrite, significantly increased the yield of spinach.

In the first experiment, seeds were soaked in an aqueous nanoparticle’s suspension and sown in the field. Seeds treated with water were used as control. The crop yields from iron-pyrite nanoparticle - treated seeds and control seeds were evaluated. The plants developed from nanoparticle - treated seeds exhibited significantly broader leaf morphology, larger leaf numbers, increased biomass; along with higher concentration of calcium (Ca), manganese (Mn) and zinc (Zn) in the leaves as compared to control.

To reduce the concentration of H_2_O_2_ generated during seed treatment with aqueous FeS_2_ nanoparticle’s suspension nano-ceria oxide CeO_2_ was added to the suspension. It was found that application of FeS_2_ resulted in obtaining leaves with increased chlorophyll and carbohydrate content which additionally were bigger than in the case of treatment with water (control), nano-CeO_2_ or FeS_2_ + CeO_2_. It was found that while nano-FeS_2_ hastened the germination and functioned as a seed vigor enhancer, nano-CeO_2_ had an opposite effect. The obtained results can be used to accelerate or delay germination, manipulate the weed population, store seeds in critical conditions and during other agro-technical treatments^22^.

The use of iron oxide nanomaterials in environmental protection technology has gained a lot of attention due to their unique properties: very small size, high surface area to volume ratio, surface modifiability, enhanced magnetic properties and excellent biocompatibility^23^. Celebi *et al*.^24^ used zero-valent iron nanoparticles (nZVI) to remove Ba^2+^ ions and Boparai *et al*.^25^ for removing Cd^2+^ ions, respectively. In order to remove water pollution with Cr^6+^, amorphous FeB alloy modified magnetite nanocomposites (Fe_3_O_4_-FeB) were used^26^. The removal of Pb^2+^ and Cd^2+^ traces from milk, human urine and blood plasma by selective ionic liquid ferrofluid in dispersive solid phase extraction was carried out by Ramandi and Shemirani^27^. Li and Zhang^28^ showed that Fe-NPs act as a sorbent and reductant when used to sequestrate Ni^2+^ in water.

For the removal of Pb^2+^ from the aqueous solution, synthesized kaolin-supported nanoscale zero-valent iron (K-nZVI)^29^ was used, which was also effective in removing Ni^2+^, Cd^2+^, Pb^2+^ (98.8%) and Cr (99.8%) from galvanic sewage.

Ge *et al*.^30^ used Fe_3_O_4_ polymer-modified magnetic nanoparticles to remove heavy metal ions (Cd^2+^, Zn^2+^, Pb^2+^ and Cu^2+^) from an aqueous solution.

Surface-active magnetic Fe_3_O_4_-NPs showed superparamagnetic behavior and a high effectiveness in removing toxic metal ions (Cr^3+^, Co^2+^, Ni^2+^, Cu^2+^, Cd^2+^, Pb^2+^ and As^3+^) and bacteria from contaminated water^31^ Badruddoza *et al*.^32^ produced carboxymethyl-β-cyclodextrin-modified Fe_3_O_4_ nanoparticles (CMCD-MNPs) to remove Cu^2+^ ions from the aqueous solution.

Bimetallic iron nanoparticles possess significantly better physical and chemical properties (including magnetism) and the ability to reduce various metals as compared to mono-metallic Fe-NPs^33^.

Ferromagnetic carbon-coated Fe-NPs showed the ability to remove more than 95% of Cr^6+^ in wastewater by means of physical adsorption on coating^34^ containing certain amount of a carboxylic functional group. Wang *et al*.^35^ developed a novel magnetic nanocomponent (core-shell) Fe_3_O_4_@SiO_2_ to remove heavy metal ions from aqueous media.

Studies on the use of Fe_2_O_3_-NPs for the selective removal of toxic metals (Cr^6+^, Cu^2+^ and Ni^2+^) from industrial wastewater have shown that the adsorption process is solution pH-dependent^36^. Liu *et al*.^37^ used Fe_3_O_4_-NPs coated with humic acid (Fe_3_O_4_/HA), to remove more than 99% of Hg^2+^ and Pb^2+^ and more than 95% of Cu^2+^ and Cd^2+^ from water. Savina *et al*.^38^ developed nanocomposite materials in which Fe-NPs were embedded in the walls of a macro-porous polymer. They showed excellent ability to remove trace concentrations of As^3+^ from the solution. Tang *et al*.^39^ created iron doped ordered mesoporous carbon (Fe/CMK-3) to remove Cr^6+^ (97%).

Corredor *et al*.^40^ used NPs to the targeted delivery of nourishing substances to treat plant diseases. The movement of Fe- and Cu-NPs in plant cells of pumpkin (*Cucurbita pepo*) was discovered.

Constant and variable magnetic and electric fields, ionizing radiation, microwave and laser radiation^41–43^ as well as magnetized water^44^ were frequently used for pre-sowing seed stimulation. Positive influence on germination and improvement of crop yield from seeds treated with electromagnetic (ELM) fields has been found^45^. According to Binhi^46^, the magneto-biological effects in a living cell are reproducible when the so-called “electromagnetic window” coincides with the “physiological” window. Positive or negative effects can be observed depending on the intensity, frequency, amplitude and the exposure time to field operation.

García Reina *et al*.^47^ suggested that the magnetic field interacts with ionic currents in the embryo’s cell membrane and, as a result of changes in ion concentration and osmotic pressure, regulates the penetration of water into seeds. Magnetic field can also affect the permeability of ion channels in the membrane and therefore the transport of substances into cells, it can enhance the formation of free radicals and induce changes in the concentration of hormones, enzymes as well as changes in DNA synthesis or transmission^48^. Under the influence of the magnetic field, the external electron shells of the water molecules and dissolved ions can became polarized. This can result in the changes of the conditions of ion hydration, which can serve as crystallization nuclei. Interaction of magnetic field with water in plant material can result in an increase in electrical conductivity and a decrease in the surface tension of water. Such an effect may be due to the Lorenz’s force exerted on the charged particles (ions) in water, which causes their temporary polarization corresponding to the external magnetic field^49,50^.

Research is rare in which seeds are exposed in magnetic fields throughout the period of germination and growth until seedlings are achieved^51^. Mroczek-Zdyrska *et al*.^52^ during the 14 day vegetation period exposed lupine seeds and emerging seedlings to constant magnetic field of 130 mT induction. The influence of a variable magnetic field with an induction of 0.2 mT and frequencies of 16 and 50 Hz on the seeds of narrow-leafed lupine (*Lupinus angustifolius* L.) was also investigated. Variable magnetic field for both frequencies reduced the content of chlorophyll and carotenoids in leaves^53^.

The analysis of the available literature showed that there were no reports on the impact of ferromagnetic particles on the germination of seeds and the growth of plants in the presence of constant magnetic fields. Not many reports exist concerning the concentrations of selected elements in the seeds and seedlings subjected to magnetic fields.

The aim of the study was to investigate the effect of aqueous solution of iron nanoparticles on the germination of sunflower *Heliantus annuus* L. seeds, the growth of seedlings and the content of selected elements in seedlings. The influence of constant magnetic fields on the above processes in systems with nanoparticles and without nanoparticles was investigated.

## Results

The description of samples used in the experiment is presented in Table 1. The results of experiments are presented as relative values showing the effects of the used factors on the parameters of seedlings and the content of elements. The effect of magnetic field was calculated against samples K-0-500 and K-0-50, respectively. The effect of nanoparticles was shown in relation to the corresponding control samples P-5-0, P-25-0 and P-120-0. The synergistic effects from the combined factors were calculated against the K-0-0 control sample.

**Table 1:** Description of the samples used in the experiment.

Magnetic field induction (mT)	Content of Fe-NPs (ppm)	Code
5	0	P-5-0
5	50	P-5-50
5	500	P-5-500
25	0	P-25-0
25	50	P-25-50
25	500	P-25-500
120	0	P-120-0
120	50	P-120-50
120	500	P-120-500
0	0	K-0-0
0	50	K-0-50
0	500	K-0-500

Additionally, for samples P-5-0, P-25-0 and P-120-0 (no Fe-NPs), the effect of the magnetic field was determined against K-0-0 sample and for samples K-0-500 and K-0-50 (no magnetic field) the effect of nanoparticles was estimated with relation to K-0-0 control sample.

The influence of Fe-NPs and magnetic field on sunflower seedlings is presented in Table 2. The highest germination capacity *G* was found for samples with no Fe-NPs (81–94%) and the lowest for those containing NPs at a concentration of 50 ppm for all magnetic field inductions as well as for the control sample K-0-50.

**Table 2:** Parameters of germination and water content of sunflower seedlings for different the magnetic field inductions and various concentrations of Fe-NPs.

	P-5-0	P-5-500	P-5-50	P-25-0	P-25-500	P-25-50	P-120-0	P-120-500	P-120-50	K-0-0	K-0-500	K-0-50
	5 mT, Fe-NPs = 0	5 mT, 500 ppm	5 mT, 50 ppm	25 mT, Fe-NPs = 0	25 mT, 500 ppm	25 mT, 50 ppm	120 mT, Fe-NPs = 0	120 mT, 500 ppm	120 mT, 50 ppm	Control, Fe-NPs = 0	Control, 500 ppm	Control, 50 ppm
G (%)	81.7 ± 13.6	73.4 ^C^ ± 5.8	64.5 ± 20.4	90.8 ± 3.6	82.2 ± 10.7	51.9 ^C^ ± 25.7	82.2 ± 6.9	73.3 ^C^ ± 8.8	69.7 ± 23.6	94.7 ± 6.5	84.4 ± 11.7	67.8 ± 33.4
H_SS_ (%)	89.1 ± 4.9	82.2 ^C^ ± 2.3	86.8 ± 5.8	92.3 ± 2.2	84.1 ± 4.1	84.5 ± 8.2	90.2 ± 1.9	83.8 ± 35	84.5 ± 4.7	93.5 ± 0.9	84.04 ± 4.19	85.4 ± 6.3
W_SS_ (g)	0.309 ± 0.064	0.211 ^C^ ± 0.019	0.277 ± 0.041	0.347 ± 0.042	0.234 ^C^ ± 0.025	0.276 ± 0.038	0.293 ± 0.039	0.236 ^C^ ± 0.022	0.253 ^C^ ± 0.062	0.314 ± 0.051	0.247 ± 0.035	0.261 ± 0.026
Values in relation to samples K-0-50 and K-0-500 (Effect of nanoparticles)												
G_REL_	0.862	0.869	0.951	0.959	0.974	0.766	0.868	0.869	1.029	—	—	—
H_REL SS_	0.961	0.979	1.016	0.987	1.0	0.990	0.964	0.997	0.989	—	—	—
W_REL SS_	0.984	0.860	1.058	1.106	0.954	1.058	0.933	0.962	0.972	—	—	—
Values in relation to sample K-0-0 (Effect of nanoparticles and magnetic field)												
G_REL_	0.863	0.775	0.681	0.959	0.868	0.548	0.868	0.774	0.736	—	0.891	0.715
H_REL SS_	0.961	0.879	0.928	0.987	0.899	0.904	0.964	0.896	0.903	—	0.899	0.913
W_REL SS_	0.984	0.672	0.882	1.105	0.745 ^C^	0.879	0.933	0.752	0.806	—	0.787	0.831
Values in relation to samples P-5-0, P-25-0 and P-120-0 (Effect of magnetic field)												
G_REL_	—	0.898	0.789	—	0.905	0.572	—	0.892	0.848	—	—	—
H_REL SS_	—	0.923	0.974	—	0.911	0.915	—	0.929	0.937	—	—	—
W_REL SS_	—	0.683	0.896	—	0.674	0.795	—	0.805	0.863	—	—	—

The mean values have been determined in relation to the appropriate control. For all control samples B = 0.

G - germination capacity, H_SS_ - mean water content, W_SS_ - wet mass od single seedling, G_REL_- relative germination, H_REL SS_ - relative water content, W_REL SS_ - Relative mass of a single seedling. Statistical significance occurred only for the combined effect of Fe-NPs and the magnetic field and is marked with C.

The highest mean water content H_*ss*_ was found in seedlings without Fe-NPs (89–94%) for all inductions of magnetic field and the K-0-0 sample. The lowest water content occurred in the remaining samples (82–86%). A similar effect was observed for the mass of single seedling W_*ss*_. *G* parameter was lower in samples without nanoparticles but higher for samples containing 500 ppm of Fe-NPs as compared with 50 ppm Fe-NPs. H_*ss*_ and W_*ss*_ in the case of samples containing nanoparticles was also lower than in that without NPs. These parameters were lower at samples containing 500 ppm of NPs than for that with 50 ppm NPs.

In the case of relative values, for most of the samples subjected to magnetic field, *G*_*REL*_ and *H*_*RELSS*_ were lower than the control regardless of field induction and Fe-NPs concentration (between 0.9–1.0 - Table 2).

*G*_*REL*_ below 1, indicates lower germination capacity for samples subjected to magnetic field apart from the P-120-50 sample. *H*_*REL SS*_ above 1 were obtained only for samples P-5-500 (increase by 5.8%), P-25-0 (10.6%) and P-25-500 (5.8%). For other samples, the effect of all the factors used (magnetic field and Fe-NPs) produced values lower that for the respective control.

In the case of parameters determined in relation to the K-0-0 sample (the effect of combined factors), all relative parameters – *G*_*REL*_, *H*_*REL SS*_ and *W*_*REL SS*_ were lower than 1 but were the highest for samples without Fe-NPs. The exception was the *W*_*SS*_ parameter for sample P-25-0. *G*_*REL*_ was higher for the sample with 500 ppm Fe-NPs than for 50 ppm Fe-NPs samples for all magnetic field inductions. *H*_*REL SS*_ and *W*_*REL SS*_ were higher for a sample containing 50 ppm Fe-NPs as compared with sample containing 500 ppm Fe-NPs - unlike in the case of *G*_*REL*_.

The influence of the applied factors on the concentration of elements in sunflower seedlings is presented in Table 3. Elements are organized in a way that at the top of the table macro elements are placed followed by microelements and toxic elements. It is impossible to assign some of the elements to microelements or toxic elements because the biological effects depend strongly on their concentration.

**Table 3:** Concentration of the elements in sunflower seedlings (ppm). Possibly trivalent elements are marked with (III).

Element	P-5-0	P-5-500	P-5-50	P-25-0	P-25-500	P-25-50	P-120-0	P-120-500	P-120-50	K-0-0	K-0-500	K-0-50
	5 mT, Fe-NPs = 0	5 mT, 500 ppm	5 mT, 50 ppm	25 mT, Fe-NPs = 0	25 mT, 500 ppm	25 mT, 50 ppm	120 mT, Fe-NPs = 0	120 mT, 500 ppm	120 mT, 50 ppm	Control, Fe-NPs = 0	Control, 500 ppm	Control, 50 ppm
Ca	2223 ^P^	1012^FCN^	1320^FCN^	1323 ^P^	1072^FCN^	1264^FC^	1242 ^P^	942^FCN^	1032^FCN^	1802	945 ^M^	1102 ^M^
K	13090 ^P^	11065^CN^	10960^FC^	12365 ^P^	10880^FCN^	10930^FCN^	12715 ^P^	11185^FCN^	11360 ^C^	12590	10510 ^M^	10670 ^M^
Mg	5150 ^P^	4839^FCN^	4861^FCN^	4854 ^P^	4633^CN^	4871 ^C^	4850 ^P^	4774 ^C^	4766 ^C^	5295	4644 ^M^	4813 ^M^
Na	44.3 ^P^	143.8^FCN^	113.1^FCN^	36.4 ^P^	97.1^FCN^	160.9^FCN^	39.2 ^P^	123.5^FC^	140.6^FCN^	39.0	112.1 ^M^	187.2 ^M^
P	14510 ^P^	12305^CN^	13210^CN^	15030 ^P^	12280^CN^	13190^CN^	14885 ^P^	12565^CN^	13230^CN^	16635	12270 ^M^	13170 ^M^
S	3344 ^P^	3239^CN^	3335^FN^	3136 ^P^	3215^CN^	3434^FN^	3147 ^P^	3206 ^C^	3399^FCN^	3148	3236 ^M^	3283 ^M^
Cu	23.6 ^P^	22.9^FCN^	22.9^FCN^	17.9 ^P^	23.0^FCN^	24.0^FCN^	18.1 ^P^	23.1^FCN^	23.5^FCN^	26.9	22.1 ^M^	22.4 ^M^
Fe (III)	200.4 ^P^	68.1^FCN^	69.1^FCN^	174.7 ^P^	71.4^FCN^	73.6^FCN^	162.3 ^P^	81.2^FCN^	68.5^FCN^	181.7	67.9 ^M^	64.3 ^M^
Mn (III)	32.67 ^P^	25.74^FCN^	27.95^FCN^	32.05 ^P^	26.63^FCN^	30.25^FCN^	31.42 ^P^	26.26^FCN^	27.13^CN^	33.28	27.47 ^M^	27.79 ^M^
Mo	1.938 ^P^	0.474 ^N^	0.499 ^N^	1.528 ^P^	0.485 ^N^	0.478 ^N^	1.461 ^P^	0.534^FN^	0.503 ^N^	1.575	0.471	0.444
Ni (III)	6.78 ^P^	4.82^FCN^	5.72^FCN^	21.08 ^P^	4.52^FCN^	5.46^FCN^	14.07 ^P^	4.90^FCN^	5.25^FCN^	28.67	4.90 ^M^	4.84 ^M^
Se	6.84 ^P^	0.91 ^N^	0.88 ^N^	2.85 ^P^	0.87 ^N^	0.87 ^N^	3.01	0.89 ^N^	0.91 ^N^	5.12	0.88	0.85
Zn	66.0 ^P^	72.3^FCN^	70.4^FCN^	72.0 ^P^	61.7^FCN^	71.9^FCN^	65.3 ^P^	70.6^FC^	69.5^FCN^	63.8	61.8 ^M^	64.1 ^M^
Cr (III)	36.24 ^P^	0.90^CN^	0.81^CN^	32.66 ^P^	0.87^CN^	0.89^CN^	32.61 ^P^	0.88^CN^	0.93^FCN^	34.99	0.85 ^M^	0.85 ^M^
Al (III)	123.75 ^P^	3.57^CN^	4.88^FCN^	44.75 ^P^	3.00^FCN^	4.35^FCN^	56.97 ^P^	2.97^CN^	2.96^CN^	60.09	3.39 ^M^	3.07 ^M^
Sr	1.16 ^P^	2.02^FCN^	4.14^FCN^	1.09 ^P^	2.09^FCN^	2.60^FCN^	1.09 ^P^	1.79^FCN^	2.17^FCN^	1.17	1.80 ^M^	2.96 ^M^
Cd	0.666 ^P^	0.676^FC^	0.771^FCN^	0.615	0.742^CN^	0.788^FCN^	0.453 ^P^	0.716^FN^	0.726^CN^	0.536	0.757 ^M^	0.700
Hg	0.774 ^P^	0.175 ^N^	0.132^FN^	0.729	0.176	0.131	0.727 ^P^	0.170 ^N^	0.136	0.780	0.152	0.143
Pb	2.58 ^P^	0.63^CN^	1.33^FC^	2.93 ^P^	0.47^CN^	0.74^FCN^	2.42 ^P^	0.53^CN^	0.77 ^C^	2.60	0.44 ^M^	1.06 ^M^

Statistical significance for the effect of the magnetic field (in relation to K-0-500 and K-0-50 respectively)-F, for the effect of Fe-NPs (against P-5-0, P-25-0 or P-120-0, respectively) - N, for the effect of Fe-NPs and magnetic field (with respect to K-0-0)-C; the effect of Fe-NPs on K-0-500 and K-0-50 (relative to K-0-0)- M, the effect of magnetic field for samples P-5-0, P-25-0 and P-120-0 (relative to K-0-0)- P.

The mostly pronounced increase in the concentration occurred for Ca (samples P-5-0: 1.23 times and P-5-50: 1.2 times) and Na (2.56–4.8) and a decrease in Ca (1.2–2.2 times; the highest for 500 ppm), K (1.13–1.2) and P (1.13–1.19). In the case of combined factors (magnetic field and Fe-NPs), there was an increase for Na (1.14–4.8) and a decrease in Ca (1.34–1.9), P (1.12–1.36) and K (1.13–1.18). The effect of the increase in the content of Zn (1.14 times for P-120-500), Mo (1.23 for P-5-0), Ca (1.2–1.23 for 5 mT and for both Fe-NPs concentrations) and Al (1.4–1.6 for 5 mT and 25 mT) in the seedlings upon magnetic field exposition was observed.

The relative decrease in Al (1.4 for P-25-50) was observed accompanied with an increase in Pb (1.13–1.43 mainly for B = 5 mT and 25 mT) and a decrease for Pb (1.4 for P- 120-500). It should be noted that for the majority of samples, the influence of the magnetic field was slight, and the relative decrease in the content of elements mainly concerns samples without Fe-NPs.

In the case of the influence of Fe-NPs on the concentration of examined elements, the relative decrease in all samples was found for Ni (1.2–4.7 times, max for K-25-500), Mo (2.7–4.1 max for 5 mT), Mn (1.6–1.27), Fe (2–2.9 max for P-5-500), Cu (1.3–1.03) and Al (10.3–34.6 max for 5 mT of both concentrations Fe-NPs).

The presence of Fe-NPs resulted in the decrease in Se concentration for all samples (range of 3.3–7.8 times; max for the magnetic field induction - 5 mT and both Fe-NPs concentrations). The decrease was also observed for P (1.18–1.36) and K (1.12–1.2). In the case of toxic elements, the decrease occurred for Hg (5.15–5.9 fold; max for 50 ppm of Fe-NPs), Cr (35–45 times; max for induction of 5 mT and 0 mT) and Pb (1.9–6.2; max for 500 ppm of Fe-NPs). The relative increase for all samples in the case of Cu (1.3 max for 25 mT), Na (2.56–4.8 max for 50 ppm) as well as for toxic elements Cd (1.16–1.6) and Sr (1.6–3.6 max for P-5-50) was observed.

The analysis of the effect of combined factors (Fe-NPs and magnetic field) for all samples showed the decrease for the following elements: Ni (1.36–6.3 times; max for Fe-NPs concentration 500 ppm), Mo (3–3.55), Mn (1.1–1.29), Fe (1.12–2.8; max for B = 0), Cu (1.14–1.22), Al (1.3 for P-25-0 to 20.3), Se (1.7–6 the majority within the limits of 6), P (1.15–1.36), Na (2.5–2.9), K (1.13–1.2) and Ca (1.4–1.9). For the toxic elements, the decrease occurred for Cr (37–43 fold), Hg (4.6–5.9 fold; max for all samples with Fe-NPs  concentration of 50 ppm) and Pb (1.96–5.9 times; max for 500 ppm). The following relative increase rates values for all the examined samples were found: Na (2.9–4.8), Mg (1.1–1.14), and Al (2 for P-5-0). The relative increase rates for toxic elements were in the range of 1.17–4.14 for Sr and between 1.15–1.4 for Cd.

For samples P-5-0, P-25-0 and P-120-0 (no Fe-NPs) as well as K-0-500 and K-0-50 (no magnetic field) analyzed with respect to K-0-0 magnetic field produced the increase of the content of the following elements: Zn, S, Na, Pb (range between 1.1–1.15), Cd (1.24) and Al (2.06). The relative decrease of Ni concentration in the range of 2.04–4.23 as well as Fe, Al, Pb, Cd in the range between 1.01–1.04, Se (1.8) and Al (1.34) were observed.

In most cases, the effect of the magnetic field was insignificant - at a level of a few to a dozen or so percent. With the increase of the magnetic field induction, the number of elements which concentrations decreased in relation to K-0-0 control increased.

The presence of Fe-NPs resulted in an increase of Cd (rates between 1.31–1.41), Sr (1.54–2.53) and Na (2.87–4.8; higher for K-0-50). A decrease in concentration was observed for Al (17.73–19.57), Fe (2.68–2.83), Cr (41.16), Ni (5.62–5.85), Pb (2.45–5.13), Hg (5.13–5.45), Mo (3.34–3.55) and Se (5.82–6.02). Interestingly higher decrease was found for K-0–500 as compared to K-0-0. Hg concentration was close to detection limit of the method used, hence for the majority of cases its change was not statistically significant (Table 3). Interestingly, Fe content in seedlings germinating and growing in the presence of nanoparticles was lower than in the control sample without Fe-NPS (Table 3).

Table 4 shows the concentrations of elements in aerial parts of sunflower seedlings growing in soil or on blotting paper as well as in sunflower seeds. No Fe-NPs were used.

**Table 4:** The content of elements and the relative factor S of its increase or decrease for seeds and seedlings growing in soil or on blotting paper.

Element	Seed (ppm)	Soil (ppm)	Blotting paper (ppm)	S_SOIL/SEEDS_	S_PAPER/SEEDS_	S_SOIL/PAPER_
Ca	1418 ± 3	18130^SC^ ± 62	4219 ^P^ ± 7	**12.79**	**2.98**	**4.29**
K	11640 ± 65	64 750^SX^	18 820 ^P^	**5.56**	**1.62**	**3.44**
Mg	2337 ± 14	6852^SC^ ± 90	6113 ^P^ ± 59	**2.93**	**2.62**	**1.12**
Na	235 ± 4	1415^SC^ ± 4	582.2 ^P^ ± 3	**6.02**	*2.43*	**2.43**
P	951 ± 42	11250^SC^ ± 138	13590 ^P^ ± 247	**1.18**	**1.43**	**0.83**
S	2175 ± 6	10810^SC^ ± 172	5025 ^P^ ± 37	**4.97**	**2.31**	**2.15**
Cu	13.69 ± 0.05	25.09^SC^ ± 0.41	26.96 ^P^ ± 0.19	**1.83**	**1.97**	**0.93**
Fe (III)	136.2 ± 0.8	741^SC^ ± 11	276.4 ^P^ ± 2.4	**5.44**	**2.03**	**2.68**
Mn (III)	20.41 ± 0.04	74.98^SC^ ± 1.06	52.59 ^P^ ± 0.45	**3.67**	**2.58**	**1.43**
Mo	0.2 ± 0.03	2.555^SC^ ± 0.031	0.704 ^P^ ± 0.021	**12.76**	**3.52**	**3.63**
Ni	3.166 ± 0.866	6.368^SC^ ± 0.128	6.899 ^P^ ± 0.029	**2.01**	**2.18**	**0.92**
Se	1.608 ± 0.461	1.789 ^C^ ± 0.475	2.787 ^P^ ± 0.115	**1.11**	**1.73**	*1.56*
Zn	27.88 ± 0.05	119.2^SC^ ± 1.9	98.81 ^P^ ± 0.69	**4.27**	**3.54**	**1.21**
Cr (III)	89.19 ± 0.46	19.52^SC^ ± 0.34	9.63 ^P^ ± 0.05	*4.56*	*9.26*	**2.03**
Al (III)	74.24 ± 1.79	346.7^SC^ ± 5.61	105 ^P^ ± 0.76	**4.67**	**1.41**	**3.30**
Sr	1.938 ± 0.006	167.6^SC^ ± 1.02	40.42 ^P^ ± 0.21	**86.48**	**20.85**	**4.15**
Cd	0.468 ± 0.008	0.946^SC^ ± 0.011	0.946 ^P^ ± 0.013	**2.02**	**2.02**	**0.99**
Hg	0.13 ± 0.04	n.d.	n.d.	*—*	*—*	**—**
Pb	3.5 ± 0.6	1.75 ± 0.49	0.729 ^P^ ± 0.287	*2.0*	*4.81*	**2.39**

The measurement involved a large amount of 7-day seedlings (over 100 pcs). The composition of the seeds was evaluated for seeds with a seed coats.

Increase - bold digits, decrease - italicized digits. Trivalent elements marked with (III). Labels: S_SOIL/SEEDS_ - relative content for seedlings growing in soil compared to seed composition, S_PAPER/SEEDS_ - Relative content for seedlings growing on blotting paper compared to seed composition, S_SOLI/PAPER_ - Relative content for seedlings growing in soil and on paper. Statistical significance: S - for the relation between seedlings and seeds, P - for relation between seedlings grown on paper and seeds, C - for relation between seedlings grown in soil and seedlings grown on paper, X - no data for determining the relationship, n.d. – not detected.

The increase in concentration for seedlings growing in the soil in relation to seedlings growing on paper was observed for Sr (increase rate of 4.15), Mo (3.63), Ca (4.29), Cr (2.03), Pb (2.4), K (3.44), Fe (2.68), Co (2.55) and Al (3.3). There was no decrease in the content of elements for any of the samples, which indicates that the elements were taken from the soil, not only from the seeds germinated on the paper. The increase in the concentration of elements for seedlings growing on blotting paper with respect to seeds was observed for Sr (20.85), Mo (3.52), Ba (9.22), Ca (2.98), Zn (3.54), Mn (2.6) and Mg (2.6) - Table 4. We are not able to explain that phenomenon at this stage. The decrease in elements’ concentrations was found for Cr (9.26), Pb (4.8), Co (2.16) and B (2.95-fold). The increase in the concentration of elements in seedlings growing in the soil in relation to seeds was as follows: Sr (86.48), Mo (12.76), Ca (12.79), Ba (15.93), Zn (4.27), Mn (3.7), Mg (2.9), K (5.56), Fe(3) (5.44) and Al (4.67). The decrease occurred for Pb, B (1.72) and Cr (4.57). The obtained results indicate a higher content of elements in seedlings growing in the soil than on blotting paper in relation to the composition of seeds.

## Discussion

The most important elements (macroelements) related to sprouting and growth of seedlings include Ca, Mg, K, P and Na. Metals such as cobalt (Co), copper (Cu), chromium (Cr), iron (Fe), magnesium (Mg), manganese (Mn), molybdenum (Mo), nickel (Ni), selenium (Se) and zinc (Zn) are essential nutrients (micronutrients) required for various biochemical and physiological functions. However, the content of these metals is strictly limited due to their toxic nature, which depends on their content in a living organism. The third group consists of heavy metals toxic to living organisms in almost any quantity. These include cadmium (Cd), mercury (Hg) and lead (Pb) which are priority metals for public health.

Considering reduction of germination parameters of seeds and the growth of sunflower seedlings one can conclude that it was the result of a decrease in the content of the elements associated with this process (Ca, Mg, K, P and Na). It can be explained in terms of adsorption of these elements by Fe-NPs.

Iron nanoparticles (Fe-NPs) are known for their metal binding properties in diverse environmental systems. They have been successfully used for the purification of water and food products from metals and other toxic compounds. Their action in the above cases was explained by the adsorption of metals on nanoparticles. A similar mechanism is probably responsible for the observed changes in the content of elements in sunflower seedlings after the addition of Fe-NPs in our experiment. This can be supported by the fact that other authors observed the penetration of nanoparticles into the seed and root of plants^54^.

Upon exposition of the sunflower roots (*Helianthus annuus* L.) to three different concentrations of nano-maghemite (NM: Fe_2_O_3_, γ-Fe_2_O_3_)^54^ reduction of the hydraulic root conductivity and lower nutrient uptake were observed. A decrease in root functionality for water uptake to 57% was observed with respect to control value at a dose of 50 mg∙l^−1^ and was reduced to 26% at a dose twice as high. This is consistent with our findings where smaller water content was found in samples subjected to NPs.

The concentrations of Ca, K, Mg and S in the shoots were also reduced compared to the control plants, which also resulted in the reduction of chlorophyll pigments in the plant. The above confirms the results obtained in our study. The decrease in macronutrients as Ca (mostly pronounced at 500 ppm of Fe-NPs), K, P and Na at the level of several percent upon treatment with NPs was observed. The decrease of parameters *G*_*REL*_, *H*_*REL SS*_ and *W*_*REL SS*_, indicate a negative impact on seed germination and development of seedlings (eventually no effect) for the majority of the cases when magnetic field of different induction and/or Fe-NPs at different concentrations were applied. In particular, interesting is the negative impact of the nanoparticles on control samples (samples containing Fe-NPs in relation to K-0-0). In that case, for higher concentrations of Fe-NPs in aqueous solution (500 ppm of Fe-NPs) a lower relative water content and relative mass of a single seedling was found.

Cifuentes *et al*.^55^ studied the effects of carbon-coated magnetic nanoparticles in the form of bio-ferrofluid on four plants: peas, sunflower, tomato and wheat. NPs were not detected outside the vascular tissues of the sunflower, unlike other plants. This means that the uptake of NPs by the roots of this plant is much slower than in other species. The above research showed that the sunflower had a lower radial ability to move bio-ferrofluid outside the vascular tissues, which might be the reason for low iron concentration in the plant tissue, similarly to our studies. In our case, the use of non-coated NPs (non-functionalized) was intended to facilitate their better penetration through the cell walls of the plant. Transmission electron microscopy images of wheat root cross-sections showed that Fe-NPs entered the root through the apoplastic path and were subsequently detected in the walls of the root epithelium cells^56^. A huge increment of Fe content in the wheat roots was observed. The authors did not detect NPs in the aboveground part (seedling), which would indicate that magnetite nanoparticles were not displaced by vascular tissues in wheat plants, which is opposite to the results found in our research.

López-Moreno *et al*.^57^ investigated tolerance of tomato *Lycopersicum Solanum* L. to CoFe_2_O_4_-NPs. In the above studies, seed exposure to NPs did not significantly affect the germination and growth of plants. The authors observed the absorption of Fe and Co into plant tissues and their effect on the concentration changes of Mg and Ca in the plant leaves.

Interestingly, much higher decrease in the content of trivalent and toxic elements occurred in our study for samples treated with the combined factors: magnetic field and Fe-NPs in comparison with treatment with Fe-NPs. The obtained results indicate the strengthening (synergistic) effect of the magnetic field in combination with nanoparticles. In our studies, a significant effect on the concentration of the elements is observed only for the samples treated with Fe-NPs. In the case of an increase in the content of elements effects of and magnetic field overlap only for Cd and Sr. In the case of a decrease in the content of elements in seedlings in relation to the control, the effect corresponds with the results of the Fe-NPs impact on the content of elements for all samples.

In conclusion, the analysis of agricultural crops exposed to NPs shows that they can be taken up by plants through roots or leaves^9^. However, the mechanisms of interaction between plants and NPs are still poorly understood and more research is needed on this subject, especially at the molecular level. Detailed studies to quantify the adsorption and uptake of NPs in different crops and under different growth conditions are necessary. In addition, there is an absence of legitimate soil-based studies with appropriate environmental exposure conditions.

## Conclusions

1. Magnetic fields had a small, mostly negative effect on germination process and germination parameters of sunflower seedlings. A clear negative effect of the presence of Fe-NPs in water on germination and sunflower seedling parameters was observed. Interestingly in many cases the decrease in the content of macro elements such as Ca, Mg, K, P and Na occurred (up to twofold).
2. For microelements, especially trivalent ones, the largest decrease in concentration occurred for samples subjected to combined magnetic field and Fe-NPs and exclusively for Fe-NPs (Al: up to 20-fold, greater for 500 ppm than for 50 ppm, Cr: up to 40-fold, Fe: up to 3-fold, Ni: up to 5,9 fold and Mn: up to 1.3-fold.
3. Application of the combined magnetic field and Fe-NPs as well as Fe-NPsalone resulted in a decrease in the content of toxic elements such as Hg and Pb (over 4 times).
4. The obtained results may indicate the strengthening effect of the magnetic field in combination with Fe-NPs (synergistic effect).
5. The obtained results may indicate that additional magnetization of Fe-NPs could increase the ability to adsorb selected elements in the roots and seeds of sunflower by iron nanoparticles.

Further studies on the composition of sunflower seedlings and roots treated with solutions with Fe-NPs of different concentrations are required. It would be desirable to determine the lowest concentrations (limit) at which the effects take place.

## Materials and Methods

Sunflower seeds were subjected to germination in the presence of Fe-NPs solution at two concentrations: 50 ppm and 500 ppm (0.005% and 0.05%). Samples were exposed to constant magnetic fields with induction of B = 5, 25 and 120 mT produced by electromagnets. Samples with B = 0 were used as controls. Samples consisted of 30 seeds placed on wet blotting paper in transparent PVC containers. The construction of containers ensured constant humidity for germinating seeds and growing seedlings.

Before sowing, the seeds were sterilized in 0.65% sodium hypochlorite solution (Aquapool, Poland) for about 5 min, then rinsed in distilled water and dried on blotting paper. The containers and the germination system were sterilized in the same way. The blotting paper was irradiated with a UV lamp for 2–3 hours before being placed in the containers.

The volume of the solution was approximately 300 mL, therefore no water or Fe-NPs solution were added to the seeds during the entire growth process. During the experiment, the containers were covered with a glass lid providing light access. The experiment was carried out in a vegetation room with a constant temperature of about 23 °C ± 2 °C, where the light intensity was E = 500 ± 5 lux (at the level of germinating seeds) and a photo-period amounted to 16/8 hours for a day and night, respectively. The lighting from the white LED tape was directed downwards to seeds and seedlings. For each of the combinations, three 7-days series of measurements were carried out.

Additionally, seeds were germinated in pots with universal soil based on high peat with an admixture of sand (20%) with a pH between 5.5 and 6.5 (TORFIKS, Poland). Germination process was conducted in a vegetation room under the above-described temperature and light conditions. No Fe-NPs was used.

The germination capacity of sunflower seeds was defined as a final number of seedlings after 7 days. The mass of a single seedling was determined by dividing the mass of all seedlings in sample by their number. The water content was measured gravimetrically and given as an average value (in %) for the whole sample. After weighing seedlings were dried in an oven at 90 °C for 24 h, and then ground with a knife mill. Ground samples were than subjected to the measurement of the concentration of selected elements.

The content of elements in the roots of sunflower seedlings germinated on blotting paper (hydroponic culture) was not measured because of the difficulty of separating them from the paper. The authors of the work assumed that it is not possible to obtain an Fe-NPs - free (uncontaminated) root sample. The content of elements in seedlings obtained from pot tests was also measured only for the aboveground part of seedlings and not for their roots. The purpose of this measurement was to be able to compare the composition of seedlings from hydroponic culture and seedlings from pot tests and to compare with the concentration of elements in seeds.

Magnetic nanoparticles (Fe-NPs) were synthesized according to the method described by Khalafalla and Reimers^58^ involving co-precipitation of ferric and ferrous ions Fe^2+^/Fe^3+^ in molar ratio of 1:2. For this purpose, 5 ml of ammonium hydroxide (NH_4_OH) 56% (= 28% NH_3_) was slowly added to 5 ml of an aqueous solution containing 1.2 g of FeCl_2_*4aq and 2.4 g of FeCl_3_*6aq and solution was vigorously stirred. After the addition was completed, the solution was stirred for 10 min. The pellet was washed with 10 ml of solution containing 0.5 ml NH_4_OH and 9.5 ml of water and after about 15 min. decanted. The above procedure was repeated four times. The mass of the obtained nanoparticles was determined after drying.

**Figure 1:**
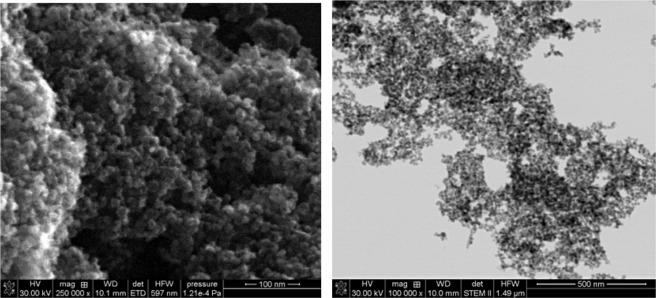
Image of the Fe_3_O_4_ nanoparticles obtained with FEI Quanta 3D FEG electron-ion microscope.

**Figure 2:**
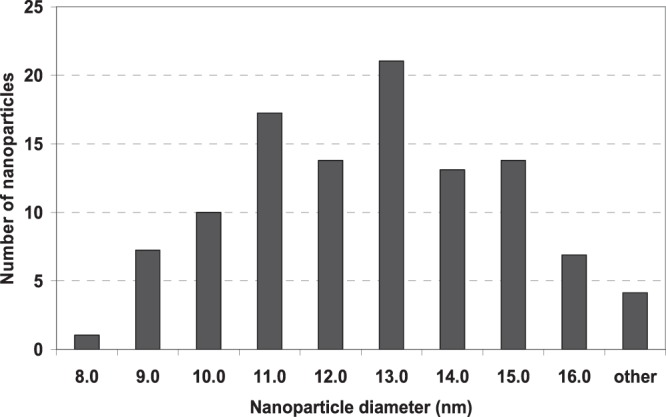
Distribution of the diameter size for the population of 140 objects of Fe-NPs (x-size in nm, y - the number of objects analyzed.

To estimate the size of the nanoparticles, a scanning electron-ion microscope FEI Quanta 3D FEG was used. The nanoparticle suspension was applied to a copper microscope mesh with a polymer film. STEM imaging was performed along with the measurement of the distribution of the size of the observed Fe-NPs (Fig. 1). The measurement consisted of adjusting the ellipse with the five-point method, and then calculating the diameter corresponding to circular objects with the same surface area as the fitted ellipses. As a result the diameter and its distribution d: 12.50 ± 4.10 nm (Fig. 2) was obtained. The uncertainty given is a doubled standard deviation for the population of 140 objects tested.

The concentration of elements was measured by the ICP-OES method according to the procedure published in Sujak *et al*.^59^.

Statistical analysis was performed using ANOVA software and the Tukey test at the significance level of p < 0.05.

## Data Availability

Data will be available on request.
